# Biosynthesis and genetic engineering of phenazine-1-carboxylic acid in *Pseudomonas chlororaphis* Lzh-T5

**DOI:** 10.3389/fmicb.2023.1186052

**Published:** 2023-04-24

**Authors:** Kaiquan Liu, Zhenghua Li, Xiaoli Liang, Yanpeng Xu, Yufei Cao, Ruiming Wang, Piwu Li, Ling Li

**Affiliations:** ^1^State Key Laboratory of Biobased Material and Green Papermaking (LBMP), School of Bioengineering, Qilu University of Technology (Shandong Academy of Sciences), Jinan, China; ^2^Shandong Provincial Key Laboratory of Biophysics, Institute of Biophysics, Dezhou University, Dezhou, China; ^3^Shandong Provincial Key Laboratory of Applied Microbiology, Ecology Institute, Qilu University of Technology (Shandong Academy of Sciences), Jinan, China

**Keywords:** phenazine-1-carboxylic acid, *Pseudomonas chlororaphis*, genetic engineering, non-scar deletion, fed-batch fermentation

## Abstract

Phenazine-1-carboxylic acid (PCA) is a biologically active substance with the ability to prevent and control crop diseases. It was certified as a pesticide by the Ministry of Agriculture of China in 2011 and was named “Shenzimycin.” Lzh-T5 is a *Pseudomonas chlororaphis* strain found in the rhizosphere of tomatoes. This strain can produce only 230 mg/L of PCA. We used LDA-4, which produces the phenazine synthetic intermediate trans-2,3-dihydro-3-hydroxyanthranilic acid in high amounts, as the starting strain. By restoring *phzF* and knocking out *phzO*, we achieved PCA accumulation. Moreover, PCA production was enhanced after knocking out negative regulators, enhancing the shikimate pathway, and performing fed-batch fermentation, thus resulting in the production of 10,653 mg/L of PCA. It suggested that *P. chlororaphis* Lzh-T5 has the potential to become an efficiency cell factory of biologically active substances.

## 1. Introduction

Rice, one of the world’s major crops, occupies an important position in the global food supply chain (Molina et al., 2011; Zhao et al., 2022). Worldwide, rice is often affected by serious diseases and insect pests. Although chemical pesticides, such as tricyclazole and epoxiconazole, are often used to control these pests and diseases, they cause environmental and food pollution (Padovani et al., 2006; Yan et al., 2015; Garcia-Jaramillo et al., 2016; Meng et al., 2018; Medina et al., 2021; Sefiloglu et al., 2021; Li H. et al., 2022). Therefore, there are increasing restrictions on the use of chemical pesticides. This has resulted in biological pesticides becoming a good alternative (Sharma et al., 2020; Ahmad et al., 2021). In this context, phenazine-1-carboxylic acid (PCA), a type of biological pesticide, has been registered and certified as a Chinese pesticide and is named “Shenzimycin” owing to its good efficacy against diseases (Jin et al., 2015; Karmegham et al., 2020; Li et al., 2021; Sun et al., 2021).

Currently, PCA is primarily produced by the strain *Pseudomonas aeruginosa*, despite it being an opportunistic pathogen and a major harmful bacterium present in hospitals (Du et al., 2015; Walker and Moore, 2015; Zhou et al., 2016; Petitjean et al., 2021). Compared with *P. aeruginosa*, *P. chlororaphis* not only contains the phenazine-synthesizing gene cluster p*hzABCDEFG* but it is also a safe strain and is more suitable as an engineered strain for the production of phenazine derivatives (Liu et al., 2016; Li et al., 2020).

Lzh-T5 is a *P. chlororaphis* strain normally found in the rhizosphere of tomatoes (Li et al., 2018). In addition to *phzABCDEFG*, it contains the phenazine modification gene *phzO*. PCA and 2-OH-PHZ can be detected in the fermentation product of the strain; therefore, it shows the potential to efficiently produce PCA (Li et al., 2018; Liu et al., 2021).

The shikimate pathway is a key metabolism pathway that is widely present in organisms, including *P. chlororaphis* (Liu et al., 2016; Li et al., 2020). A series of substances, such as coenzyme Q, aromatic amino acids, and phenazines, can be produced through the shikimate pathway (Talapatra and Talapatra, 2005; Liu et al., 2016). Trans-2,3-dihydro-3-hydroxyanthranilic acid (DHHA) is also an accumulation product of the shikimate pathway truncated in *P. chlororaphis*. Our previous study revealed that *P. chlororaphis* Lzh-T5 can produce DHHA (Liu et al., 2021).

In this study, we used a derivative strain of *P. chlororaphi*s Lzh-T5, LDA-4, which possess high DHHA production capacity, as the starting strain and achieved PCA accumulation as a single product through genetic engineering. Gene expression and feed-bath strategies were used to increase the PCA production by the strain, resulting in an engineered strain that produced 10.65 g/L of PCA.

## 2. Materials and methods

### 2.1. Strains and bacterial culture

Different *Escherichia coli* and *P. chlororaphis* strains were used in this study (Table 1). *E. coli* is primarily used for gene knockout vector construction and biparental hybridization, whereas *P. chlororaphis* is primarily used for the construction of engineered strains producing phenazine derivatives. *E. coli* was cultured in LB medium at 37°C, and *P. chlororaphis* was cultured in King’s B medium at 28°C. The composition of these media has been described in our previous study (Li et al., 2020). Ampicillin and kanamycin sulfate were used as antibiotics at concentrations of 100 and 50 μg/ml, respectively.

**Table 1:** Strains and plasmids used in this study.

Strains and plasmids	Relevant gene type	Reference/source
**Strains**		
*Escherichia coli* DH5α	*E. coli* F^−^Ф80*lacZ*ΔM15 Δ(*lacZYA*-*argF*) U169 *recA1 endA1 hsdR17* (r_k_^−^ m_k_^−^) *phoA supE44 thi*^−1^ *gyrA96* relA1	Lab stock
*Escherichia coli* S17-1(λpir)	res^−^ pro mod^+^ integrated copy of RP4, mob^+^, used for incorporating constructs into *P. chlororaphis*	Lab stock
*Pseudomonas chlororaphis* Lzh-T5	*P. chlororaphis* Lzh-T5 wild-type strain	Lab stock
*Pseudomonas chlororaphis* LDA-4	*phzF, pykF, psrA* and *rpeA* in-frame deletion mutant of Lzh-T5	Lab stock
*Pseudomonas chlororaphis* LDA-4-phzF	*phzF* in-frame insertion mutant of *P. chlororaphis* LDA-4	This study
*Pseudomonas chlororaphis* LDPCA-1	*phzO* in-frame deletion mutant of *P. chlororaphis* LDA-4-phzF	This study
*Pseudomonas chlororaphis* LDPCA-2	*rsmE i*n-frame deletion mutant of *P. chlororaphis* LDPCA-1	This study
*Pseudomonas chlororaphis* LDPCA-3	*parS* in-frame deletion mutant of *P. chlororaphis* LDPCA-2	This study
*Pseudomonas chlororaphis* LDPCA-4	*lon* in-frame deletion mutant of *P. chlororaphis* LDPCA-3	This study
*Pseudomonas chlororaphis* LDPCA-5	*ppsA* in-frame insertion mutant of *P. chlororaphis* LDPCA-4	This study
*Pseudomonas chlororaphis* LDPCA-6	*tktA i*n-frame insertion mutant of *P. chlororaphis* LDPCA-5	This study
**Plasmids**		
pEASY-Blunt	Blunt vector for gene coloning, Ap^r^, Kan^r^	Lab stock
pEASY-Blunt-tktA	PCR cloning amplification vector for gene *tktA*	This study
pEASY-Blunt-ppsA	PCR cloning amplification vector for gene *ppsA*	This study
pK18mobsacB	Broad-host-range gene replacement vector, *sacB*, Kan^r^	Lab stock
pK18-phzF	pK18mobsacB containing *phzF* flanking region	This study
pK18-phzO	pK18mobsacB containing *phzO* flanking region	This study
pK18-rsmE	pK18mobsacB containing *rsmE* flanking region	This study
pK18-parS	pK18mobsacB containing *parS* flanking region	This study
pK18-lon	pK18mobsacB containing *lon* flanking region	This study
pK18-ppsA	pK18mobsacB containing *ppsA* flanking region	This study
pK18-tktA	pK18mobsacB containing *tktA* flanking region	This study

### 2.2. Genetic engineering

The plasmids and primers used in this study are presented in Table 1 and Supplementary Table S1, respectively. *phzF* was restored into the genome of the LDA-4 strain through gene insertion. First, the primers for *phzF* insertion were designed according to the genome sequence of *P. chlororaphis* Lzh-T5 (Supplementary Table S1). Subsequently, *phzF* and its upstream and downstream fragment phzFUD was amplified through PCR using phzF-F1/phzF-R2 as primers and the Lzh-T5 genome as the template.

The restriction enzymes EcoRI and XbaI were used to digest the phzFUD fragment and the pK18mobSacB plasmid, respectively. After recovery through agarose gel electrophoresis, the recombinant plasmid pK18-phzFUD was obtained using T4 ligase. This plasmid was transformed into *E. coli* S17-1 (λ), resulting in the recombinant strains *E. coli* S17-1 (λ) and *P. chlororaphis* LDA-4; these strains were cultured respectively, and the pK18-phzFUD plasmid was introduced into pseudo-LDA-4 by biparental hybridization. The plasmid and the bacterial genome were double-exchanged on a King’s B medium plate containing 10% sucrose, and the *phzF*-restored strain was obtained through PCR.

Genes such as *rsmE*, *parS*, and *lon* have been knocked out from the genome of *P. chlororaphis* either individually or together, as described in our previous studies (Liu et al., 2016; Li et al., 2020).

### 2.3. Quantitative RT-PCR

Quantitative RT-PCR was performed to detect the transcriptional changes of related genes in different *P. chlororaphis* strains as described in our previous study (Liu et al., 2021). *rpoD*, a housekeeping gene of *P. chlororaphis*, was selected as the internal reference gene. The mRNA fold change was calculated using the 2^−ΔΔCt^ method (Livak and Schmittgen, 2001).

### 2.4. Fed-batch fermentation of strains

To maximize the efficacy of the engineered strains, we added the high-yielding strains LDPCA-6 to a 5-L fermenter to perform fed-batch fermentation with specific fermentation parameters according to our previous research (Li et al., 2020).

### 2.5. Separation, preparation, and detection of phenazine samples

PCA must be extracted from the fermentation broth and then detected and quantified by high-performance liquid chromatography (HPLC). We first extracted PCA from the fermentation broth using ethyl acetate and subjected it to reversed-phase C18 column (Agilent Technologies, 5 μm, 4.6*250 mm). Both 2-OH-PHZ and PCN are phenazine derivatives and exhibit similar material properties; the specific extraction and detection steps of these samples are described in our previous studies (Liu et al., 2016; Li et al., 2020).

## 3. Results

### 3.1. Restoration of *phzF* in LDA-4 resulted in the accumulation of phenazines

**Figure 1:**
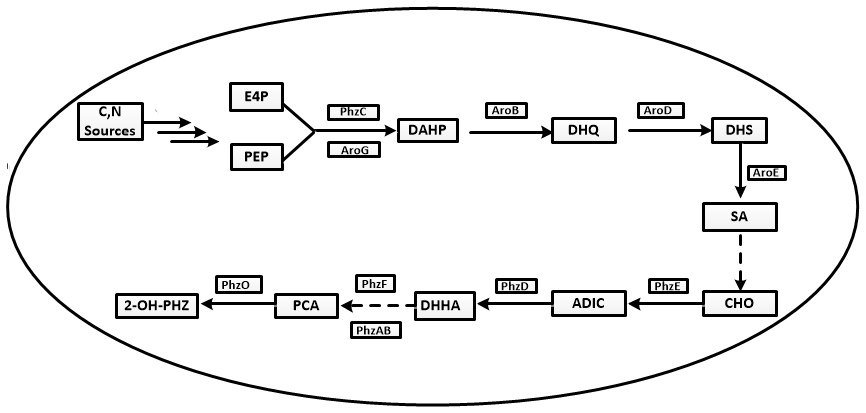
The synthetic pathway of phenazine-1-carboxylic acid and its derivatives in *Pseudomonas chlororaphis*. E4P, erythrose 4-phosphate; PEP, phosphoenolpyruvic acid; DAHP, 3-deoxy-darabinoheptulosonate-7-phosphate; DHQ, 3-dehydroquinic acid; DHS, 3-dehydroshikimic acid; SA, shikimic acid; CHO, chorismate; ADIC, 2-amino-4-deoxy branched acid; DHHA, Trans-2,3-dihydro-3-hydroxyanthranilic acid; PCA, Phenazine-1-carboxylic acid; 2-OH-PHZ, 2-hydroxyphenazine.

**Figure 2:**
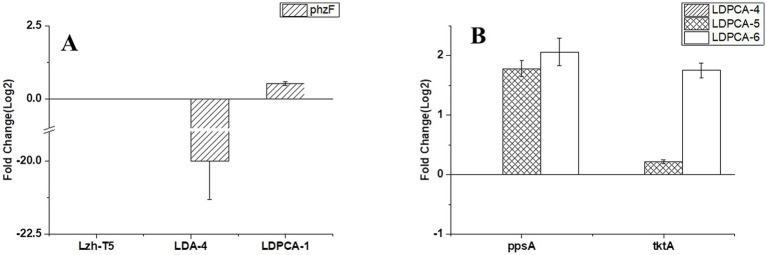
Transcriptional validation of different genes using Quantitative RT-PCR. **(A)** Transcriptional validation of phzF in different strains. **(B)** Transcriptional validation of tktA and ppsA in different strains. The data represent the means ± SD for three independent cultures.

**Figure 3:**
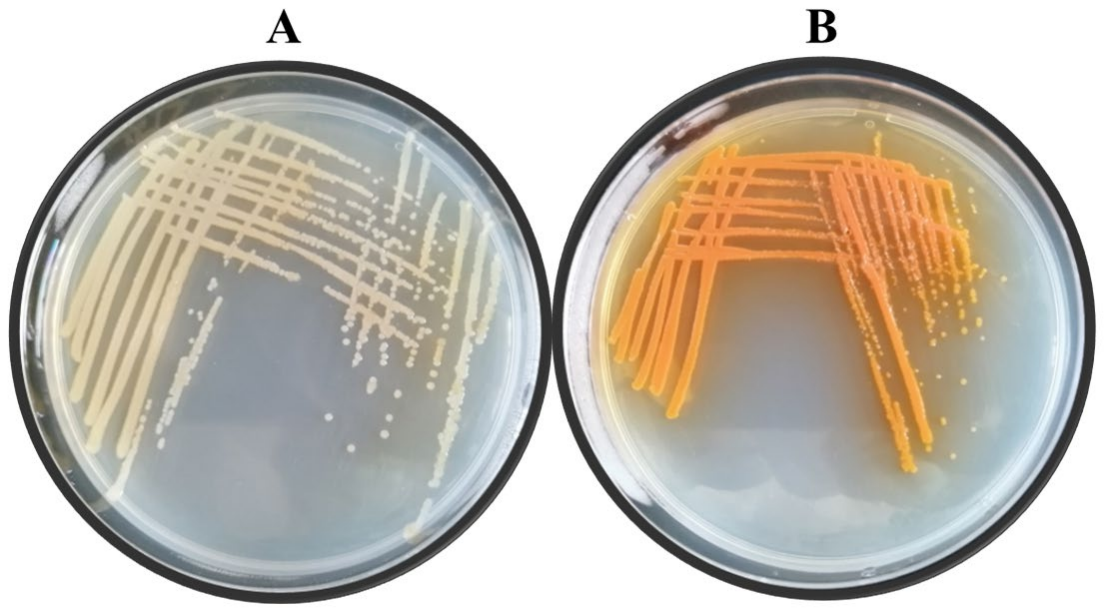
Colony morphology of different strains. **(A)** *Pseudomonas chlororaphis* LDA-4. **(B)** *Pseudomonas chlororaphis* LDA-4-phzF.

**Figure 4:**
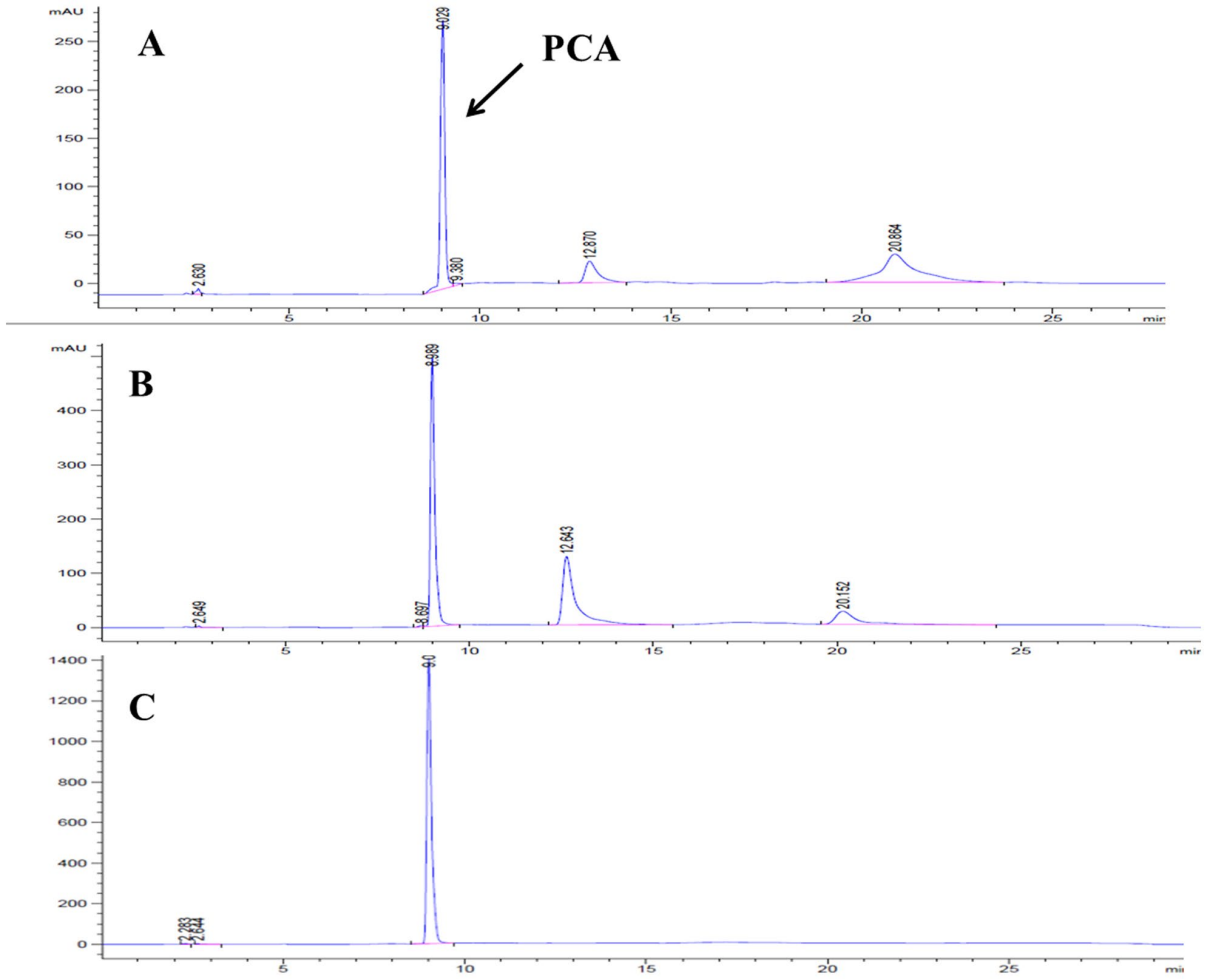
HPLC detection of fermentation broth of different strains. **(A)** *Pseudomonas chlororaphis* Lzh-T5. **(B)** *Pseudomonas chlororaphis* LDPCA-1. **(C)** *Pseudomonas chlororaphis* LDPCA-1.

**Figure 5:**
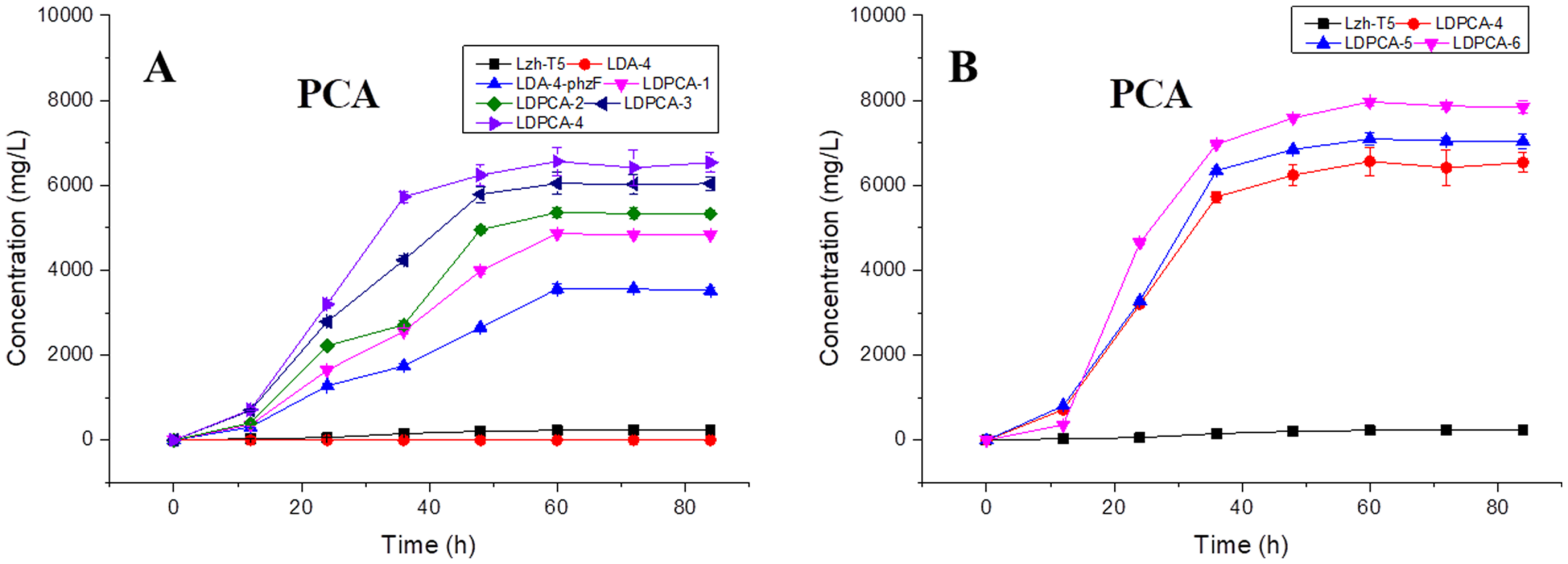
Effects of genetic modifications on PCA production. **(A)** PCA production of non-scar deletion strains. **(B)** PCA production of gene over-expression strains. The data represent the means ± SD for three independent cultures.

**Figure 6:**
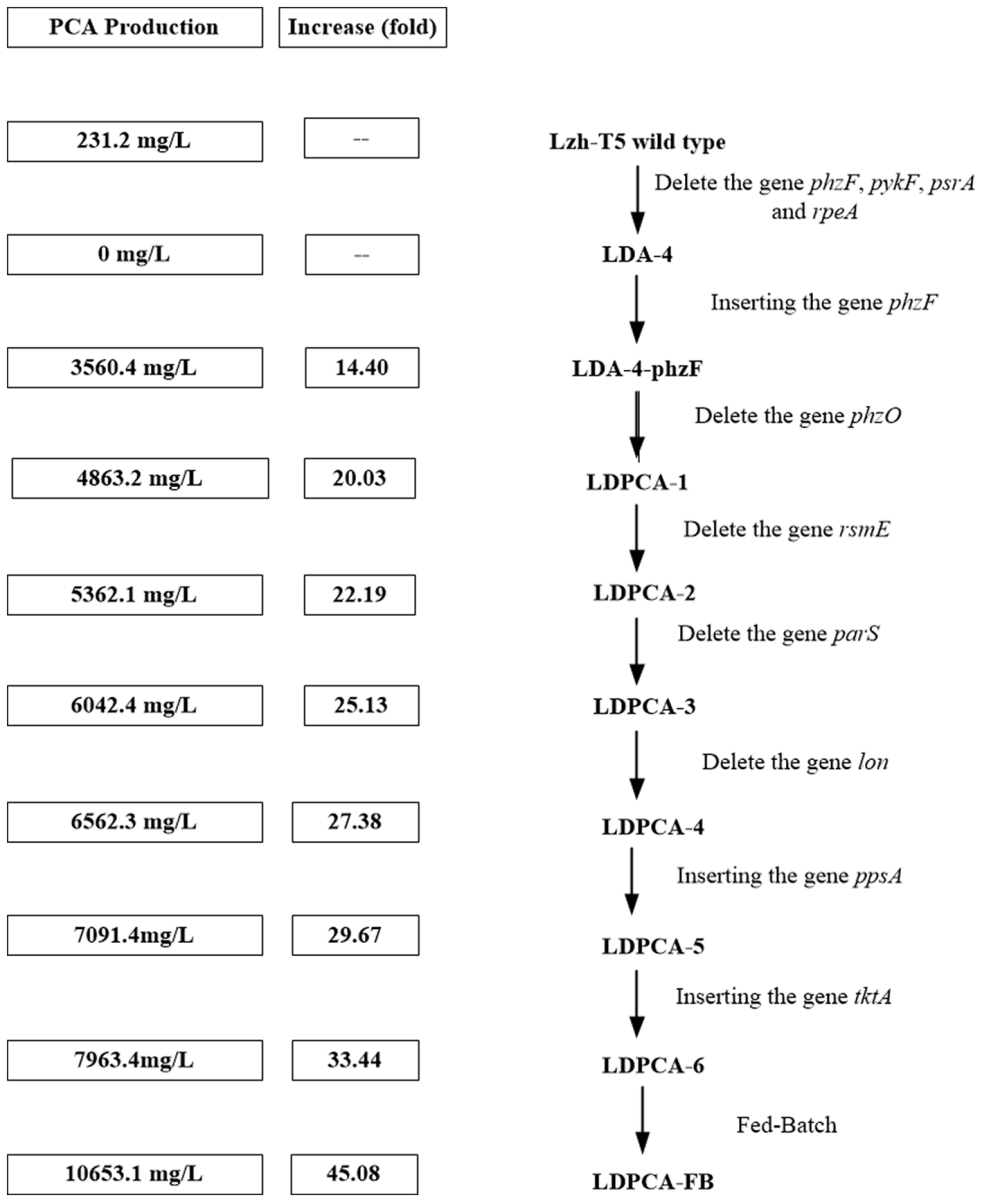
A summary of PCA production improve via genetic engineering operations in *Pseudomonas chlororaphis.*

Lzh-T5 is a *P. chlororaphis* strain selected from the rhizosphere of tomato. It is known that its genome contains the key *phzABCDEFG* gene cluster for the synthesis of phenazine derivatives. The fermentation broth of this strain in KB would turn to orange (which is the color of 2-hydroxyphenazine), with the potential to produce phenazine derivatives. In our previous research, we blocked the biosynthesis of phenazine by knocking out *phzF*, which led to the accumulation of DHHA, an important chemical intermediate. We strengthened the synthetic pathway of DHHA via genetic engineering and obtained the engineered strain LDA-4. The yield of DHHA using LDA-4 reached 5.52 g/L in the shake flask (Liu et al., 2021). In the present study, we used LDA-4 to obtain high yield of PCA. DHHA accumulation occurred in LDA-4 owing to the knockout of *phzF* in the phenazine synthesis pathway (Figure 1). We restored the function of *phzF* in the genome of LDA-4 through genetic engineering to obtain the strain LDA-4-phzF. To confirm whether *phzF* could indeed play a role, we performed RT-PCR to verify its expression level in different strains. Our results revealed increased expression of *phzF* in LDA-4-phzF compared with that in LDA-4 (Figure 2A). At 28°C, the colony of the strain turned red (Figure 3). HPLC revealed three phenazine derivative peaks in LDA-4-phzF (Figure 4). Compared with the wild-type strain Lzh-T5, the yield of phenazines was increased to 3560.4 mg/L in LDA-4-phzF (Figures 5, 6).

### 3.2. Knockout of *phzO* resulted in PCA accumulation in the strain

After genome sequencing, the phenazine-related gene *phzO* was detected in *P. chlororaphis* Lzh-T5 (Li et al., 2018). The PhzO protein encoded by *phzO* is a phenazine-modified protein that catalyzes PCA conversion into 2-OH-PCA, which then spontaneously converts into 2-OH-PHZ (Chen et al., 2014). Therefore, both Lzh-T5 and its derivative strain LDA-4-phzF produce three phenazine derivatives, PCA, 2-OH-PCA, and 2-OH-PHZ (Liu et al., 2021). In this study, we knocked out *phzO* from the genome of the LDA-4-phzF strain using traceless knockout technology to obtain the LDPCA-1 strain. The metabolites of LDPCA-1 were extracted and detected using HPLC. Only PCA was accumulated in LDPCA-1 (Figure 4), with a yield of 4,863 mg/L, which was 20.9 times higher than that in the wild-type strain Lzh-T5 (Figures 5A, 6).

### 3.3. Enhancement of PCA production by knockout of negative regulatory factors

To enhance the production of PCA, several widely occurring negative regulator genes in *P. chlororaphis* were selected for knockout. We knocked out the negative regulatory factor *rsmE* from the genome of LDPCA-1 to obtain LDPCA-2 strain. HPLC revealed that PCA production by this strain increased to 5362.1 mg/L (Figures 5A, 6). Knockout of *parS*, another negative regulatory factor in the genome of LDPCA-2, resulted in the strain LDPCA-3, which presented with an increase in PCA yield to 6042.3 mg/L (Figures 5A, 6). The engineered strain LDPCA-4 was obtained by knocking out *lon*, and the PCA yield of this strain increased to 6562.3 mg/L (Figures 5A, 6).

### 3.4. Increasing PCA production by enhancing the shikimate pathway

Phenazine derivatives in *Pseudomonas* spp. are synthesized through the shikimate pathway. Enhancing this phenazine synthesis pathway can help promote PCA production. The shikimate pathway is an important metabolic pathway in *Pseudomonas* spp., which can not only synthesize phenazines but is also the lead pathway for coenzyme Q and aromatic amino acid synthesis (Talapatra and Talapatra, 2005; Liu et al., 2016). Phosphoenolpyruvate (PEP) and erythrose 4-phosphate (E4P) are the direct synthetic precursors of the shikimate pathway (Talapatra and Talapatra, 2005). Enhancing PEP and E4P supply can enhance the shikimate pathway (Rodriguez et al., 2014). Therefore, we selected *ppsA* and *tktA,* genes that can improve E4P and PEP production (Hu et al., 2017; Li et al., 2020). Accordingly, *ppsA* was integrated at the *lon* position of LDPCA-4 via homologous integration to obtain the recombinant strain LDPCA-5. Next, *tktA* was integrated at the *parS* position of the LDPCA-5 strain to obtain the recombinant strain LDPCA-6. RT-PCR revealed that the expression levels of *tktA* and *ppsA* in LDPCA-6 and LDPCA-5 were higher than those in LDPCA-4 (Figure 2B), suggesting that the genes integrated into the genome are transcribed and play a role in enhancing the shikimate pathway. HPLC detection revealed that the PCA production of LDPCA-5 and LDPCA-6 was increased, reaching 7091.4 and 7963.4 mg/L, respectively (Figures 5B, 6).

### 3.5. Enhancing PCA production by fed-batch fermentation

**Figure 7:**
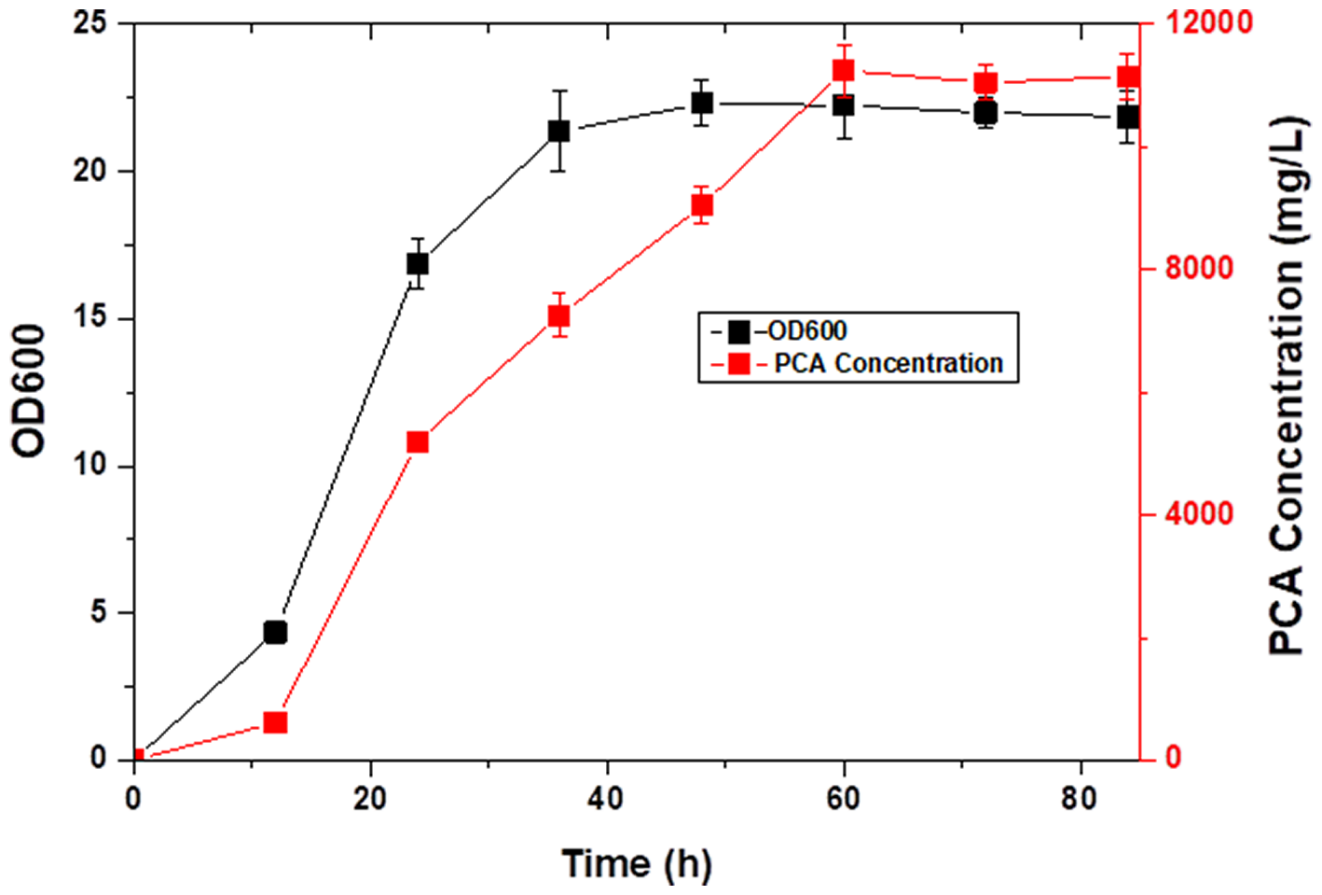
PCA production in LDPCA-6 during fed-batch fermentation.

*Pseudomonas* spp. are aerobic bacteria; therefore, shake flask fermentation often limits the maximum efficiency of bacteria owing to insufficient oxygen supply. Moreover, after a period of growth, the efficiency of bacteria is limited due to lack of nutrients. In this study, we used the fed-batch method to ferment the engineered strain to improve PCA production by the bacterial strain. The fed-batch experiments were conducted in a 5-L fermenter after activating LDPCA-6 in a shake flask. PCA was extracted from the fermentation broth during the fermentation process. HPLC detection revealed that the bacterial growth reached a stable stage at 48 h after inoculation, and PCA production continued to increase for 60 h. The maximum yield was 10,653.1 mg/L, and the DCW yield was 2286.6 mg/g (Figures 6, 7).

## 4. Discussion

Phenazine derivatives are a class of biologically active substances that can be naturally produced by the *Pseudomonas* and *Streptomyces* spp. (Chin et al., 2000; Laursen and Nielsen, 2004; Mavrodi et al., 2006; Lugtenberg and Kamilova, 2009; Bilal et al., 2017). In this study, we focused on PCA production by *Pseudomonas* spp.

The synthesis steps of PCA by *Pseudomonas* are as follows. First PEP and E4P are converted to shikimate via the shikimate pathway. Then, shikimate is converted to PCA via enzymes encoded by the conserved phenazine synthesis gene cluster *phzABCDEFG* (Figure 1).

Different phenazine modification genes are present in *Pseudomonas* spp. that convert PCA into different phenazine derivatives, such as *phzO*, which converts PCA into 2-hydroxyphenazine; *phzH*, which converts PCA into phenazine-1-carboxamide; *phzS*, which converts PCA into 1-hydroxyphenazine; and *phzS* and *phzM*, which act simultaneously to convert PCA into pyocyanin (Chin et al., 2001; Mavrodi et al., 2001; Parsons et al., 2007; Greenhagen et al., 2008; Du et al., 2013).

Lzh-T5 is a strain of *P. chlororaphis* commonly found in the rhizosphere of tomatoes in Dezhou, China. Using genome sequencing, this strain was found to contain the typical phenazine synthesis gene cluster *phzABCDEFG* and a phenazine modification gene *phzO*. After fermentation, both PCA 2-OH-PCA and 2-OH-PHZ were present in the fermentation broth. We knocked out *phzO* using a traceless knockout method, which cut off the conversion of PCA into 2-OH-PHZ. After fermentation, HPLC detection revealed that the fermentation broth could accumulate PCA alone (Figure 4).

The wild strain *Pseudomonas* Lzh-T5 can produce PCA, although the yield is very low at only 220 mg/L. In our preliminary work, we used Lzh-T5 to produce DHHA through genetic engineering and obtained a yield of 11 g/L (Liu et al., 2021). DHHA is the synthetic precursor of phenazine derivatives in *Pseudomonas* and was synthesized by *Pseudomonas* after *phzF* knockout (Liu et al., 2021). Because of the metabolic modification of the DHHA-producing strain in this study, its metabolic flux in the shikimate pathway was stronger than that in the wild-type strain Lzh-T5. In this study, we selected the high DHHA-producing strain LDA-4 to produce PCA by restoring *phzF*.

Two-component signal transduction (TCST) systems are a class of regulatory systems that are widely present in bacteria and help bacteria adapt to changes in the surrounding environment. According to previous reports, *Pseudomonas* spp. contains a large number of TCST systems, such as the GacS/GacA and rpeA/rpeB systems (Bejerano-Sagie and Xavier, 2007; Peng et al., 2018). The GacS/GacA system is a TCST system that has been previously identified in *Pseudomonas* spp. Studies have reported that the GacS/GacA system affects the production of phenazines through intracellular small RNAs and negatively regulates the production of phenazines through *rsmE* (Liu et al., 2016; Li et al., 2020). We found *rsmE* to be present in the Lzh-T5 genome, and, after knocking out *rsmE* in the LDPCA-1 strain genome, PCA production increased from 4863.2 mg/L to 5362.1 mg/L. Compared with the GacS/GacA system, the parS/parR system has recently been discovered. Studies have also reported that the parS/parR system can regulate phenazine production in *Pseudomonas* (Liu et al., 2016; Peng et al., 2018). We obtained the strain LDPCA-3 by knocking out *parS* in the LDPCA-2 genome; moreover, HPLC revealed increased PCA production (Figures 5, 6). In addition to the TCST systems that can affect the production of phenazines, some enzymes can indirectly regulate phenazine synthesis through the TCST system. Reportedly, the protease LON has been reported to be a negative regulatory factor of phenazine derivatives (Laskowska et al., 1996; Whistler et al., 2000; Wang et al., 2013; Takeuchi et al., 2014; Liu et al., 2016). And LON protease affects the production of 2-hydroxyphenazine by affecting the stability of GacA protein (Liu et al., 2016; Li et al., 2020). After detection, LON was found in *P. chlororaphis* Lzh-T5. Our results indicate that LON negatively regulates PCA production (Figures 5, 6).

In *Pseudomona*s spp., the shikimate pathway is the leading pathway for phenazine synthesis. This pathway uses PEP and E4P as direct synthetic substrates (Talapatra and Talapatra, 2005). Previous research has shown that the expression of PEP synthase (encoded by *ppsA*) and transketolase (encoded by *tktA*) can increase the PEP and E4P pools in *Pseudomonas* cells, enhancing the shikimate pathway (Liu et al., 2016). In some previous studies, genes such as *ppsA* and *tktA* were overexpressed to promote the synthesis of phenazines, achieving good yields (Liu et al., 2016; Hu et al., 2017; Li et al., 2020). However, these studies generally used plasmids as vectors for overexpression. Plasmids are generally confirmed by antibiotics to ensure their stable existence in bacteria and are easily lost in the absence of antibiotics. Furthermore, when multiple genes are expressed simultaneously, the sequence order will affect the expression effect of different genes (Juminaga et al., 2012; Liu et al., 2016). These problems are not encountered when the overexpressed gene is introduced into the genome of the host bacterium. In this study, we used the principle of homologous recombination to introduce *ppsA* and *tktA* into the genome of *Pseudomonas* for over expression. The results of RT-PCR and PCA detection showed that the introduced *ppsA* and *tktA* promoted the shikimate pathway for PCA production (Figure 2).

PCA is a secondary metabolite of *Pseudomonas* spp. and needs to be produced during the growth of *Pseudomonas* spp. (Li S. et al., 2022; Olyaei and Sadeghpour, 2022). The growth and development of bacteria can be generally divided into the lag, logarithmic growth, and extinction phases. PCA production is more favorable with *Pseudomonas* in a stable state (Liu et al., 2016; Yue et al., 2018). Compared with batch fermentation, the fed-batch fermentation medium has rich ingredients, stable oxygen supply, and relatively constant pH value, which are more conducive to the rapid reproduction of microorganisms and the production of metabolites (Li et al., 2020). Therefore, the fed-batch process is the preferred fermentation mode. Moreover, PCA produced in this study is a secondary metabolite of *Pseudomonas*, and a large amount of accumulation will lead to feedback inhibition in the bacteria. The problems of substrate inhibition and catabolite inhibition can be overcome in the fed-batch process by the continuous addition of new medium. This significantly increases the yield of the target product. In this study, we added the PCA high-yielding strain LDPCA-6 to a 5-L fermenter for fed-batch fermentation. The HPLC results showed that the bacterial growth reached a stable stage at 60 h after inoculation, and the production of PCA continued to increase for 72 h. The maximum yield was 10,653.1 mg/L, which was 33.8% higher than that using batch culture.

## Data availability statement

The original contributions presented in the study are included in the article/Supplementary material, further inquiries can be directed to the corresponding author.

## Author contributions

LL and KL conceived, designed the experiments, and drafted the manuscript. KL, ZL, XL, YX, and YC performed the experiments. KL, RW, LL, and PL analyzed the data. All authors read and approved the final manuscript.

## Funding

This work was financially supported by the Qilu University of Technology of Cultivating Subject for Biology and Biochemistry (no. 202008), the International Technology Cooperation Project from Qilu University of Technology (Shandong Academy of Sciences) (no. 2022GH026), the Shandong Provincial Key Laboratory of Biophysics (no. FWL2021065), the Natural Science Foundation of Shandong Province (nos. ZR2019BC027 and ZR2020QC044), and the National Natural Science Foundation of China (no. 31800106). The funders had no role in study design, data collection and analysis, decision to publish, or preparation of the manuscript.

## Conflict of interest

The authors declare that the research was conducted in the absence of any commercial or financial relationships that could be construed as a potential conflict of interest.

## Publisher’s note

All claims expressed in this article are solely those of the authors and do not necessarily represent those of their affiliated organizations, or those of the publisher, the editors and the reviewers. Any product that may be evaluated in this article, or claim that may be made by its manufacturer, is not guaranteed or endorsed by the publisher.

## References

[ref1] Ahmad G. Khan A. Khan A. A. Ali A. Mohhamad H. I. (2021). Biological control: a novel strategy for the control of the plant parasitic nematodes. Antonie Van Leeuwenhoek 114, 885–912. doi: 10.1007/s10482-021-01577-9, PMID: 33893903

[ref2] Bejerano-Sagie M. Xavier K. B. (2007). The role of small RNAs in quorum sensing. Curr. Opin. Microbiol. 10, 189–198. doi: 10.1016/j.mib.2007.03.009 17387037

[ref3] Bilal M. Guo S. Iqbal H. M. N. Hu H. Wang W. Zhang X. (2017). Engineering *Pseudomonas* for phenazine biosynthesis, regulation, and biotechnological applications: a review. World J. Microbiol. Biotechnol. 33:191. doi: 10.1007/s11274-017-2356-9, PMID: 28975557

[ref4] Chen M. Cao H. Peng H. Hu H. Wang W. Zhang X. (2014). Reaction kinetics for the biocatalytic conversion of phenazine-1-carboxylic acid to 2-hydroxyphenazine. PLoS One 9:e98537. doi: 10.1371/journal.pone.0098537, PMID: 24905009 PMC4048165

[ref5] Chin A. W. T. F. Bloemberg G. V. Mulders I. H. Dekkers L. C. Lugtenberg B. J. (2000). Root colonization by phenazine-1-carboxamide-producing bacterium *pseudomonas chlororaphis* PCL1391 is essential for biocontrol of tomato foot and root rot. Mol. Plant-Microbe Interact. 13, 1340–1345. doi: 10.1094/MPMI.2000.13.12.1340, PMID: 11106026

[ref6] Chin A. W. T. F. Thomas-Oates J. E. Lugtenberg B. J. Bloemberg G. V. (2001). Introduction of the *phzH* gene of *Pseudomonas chlororaphis* PCL1391 extends the range of biocontrol ability of phenazine-1-carboxylic acid-producing *Pseudomonas* spp. strains. Mol. Plant-Microbe Interact. 14, 1006–1015. doi: 10.1094/MPMI.2001.14.8.1006, PMID: 11497461

[ref7] Du X. Li Y. Zhou Q. Xu Y. (2015). Regulation of gene expression in *Pseudomonas aeruginosa* M18 by phenazine-1-carboxylic acid. Appl. Microbiol. Biotechnol. 99, 813–825. doi: 10.1007/s00253-014-6101-0, PMID: 25304879

[ref8] Du X. Li Y. Zhou W. Zhou Q. Liu H. Xu Y. (2013). Phenazine-1-carboxylic acid production in a chromosomally non-scar triple-deleted mutant *Pseudomonas aeruginosa* using statistical experimental designs to optimize yield. Appl. Microbiol. Biotechnol. 97, 7767–7778. doi: 10.1007/s00253-013-4921-y, PMID: 23636695

[ref9] Garcia-Jaramillo M. Redondo-Gomez S. Barcia-Piedras J. M. Aguilar M. Jurado V. Hermosin M. C.. (2016). Dissipation and effects of tricyclazole on soil microbial communities and rice growth as affected by amendment with alperujo compost. Sci. Total Environ. 550, 637–644. doi: 10.1016/j.scitotenv.2016.01.174, PMID: 26849328

[ref10] Greenhagen B. T. Shi K. Robinson H. Gamage S. Bera A. K. Ladner J. E.. (2008). Crystal structure of the pyocyanin biosynthetic protein PhzS. Biochemistry 47, 5281–5289. doi: 10.1021/bi702480t, PMID: 18416536

[ref11] Hu H. Li Y. Liu K. Zhao J. Wang W. Zhang X. (2017). Production of trans-2,3-dihydro-3-hydroxyanthranilic acid by engineered *Pseudomonas chlororaphis* GP72. Appl. Microbiol. Biotechnol. 101, 6607–6613. doi: 10.1007/s00253-017-8408-0, PMID: 28702795

[ref12] Jin K. Zhou L. Jiang H. Sun S. Fang Y. Liu J.. (2015). Engineering the central biosynthetic and secondary metabolic pathways of *Pseudomonas aeruginosa* strain PA1201 to improve phenazine-1-carboxylic acid production. Metab. Eng. 32, 30–38. doi: 10.1016/j.ymben.2015.09.003, PMID: 26369437

[ref13] Juminaga D. Baidoo E. E. Redding-Johanson A. M. Batth T. S. Burd H. Mukhopadhyay A.. (2012). Modular engineering of L-tyrosine production in *Escherichia coli*. Appl. Environ. Microbiol. 78, 89–98. doi: 10.1128/AEM.06017-11, PMID: 22020510 PMC3255607

[ref14] Karmegham N. Vellasamy S. Natesan B. Sharma M. P. Al Farraj D. A. Elshikh M. S. (2020). Characterization of antifungal metabolite phenazine from rice rhizosphere *Fluorescent pseudomonads* (FPs) and their effect on sheath blight of rice. Saudi J. Biol. Sci. 27, 3313–3326. doi: 10.1016/j.sjbs.2020.10.007, PMID: 33304137 PMC7715052

[ref15] Laskowska E. Kuczynska-Wisnik D. Skorko-Glonek J. Taylor A. (1996). Degradation by proteases Lon, Clp and HtrA, of *Escherichia coli* proteins aggregated in vivo by heat shock; HtrA protease action *in vivo* and *in vitro*. Mol. Microbiol. 22, 555–571. doi: 10.1046/j.1365-2958.1996.1231493.x, PMID: 8939438

[ref16] Laursen J. B. Nielsen J. (2004). Phenazine natural products: biosynthesis, synthetic analogues, and biological activity. Chem. Rev. 104, 1663–1686. doi: 10.1021/cr020473j, PMID: 15008629

[ref17] Li H. Li Y. Wang W. Wan Q. Yu X. Sun W. (2022). Uptake, translocation, and subcellular distribution of three triazole pesticides in rice. Environ. Sci. Pollut. Res. Int. 29, 25581–25590. doi: 10.1007/s11356-021-17467-6, PMID: 34850341

[ref18] Li L. Li Z. Yao W. Zhang X. Wang R. Li P.. (2020). Metabolic engineering of *Pseudomonas chlororaphis* Qlu-1 for the enhanced production of phenazine-1-carboxamide. J. Agric. Food Chem. 68, 14832–14840. doi: 10.1021/acs.jafc.0c05746, PMID: 33287542

[ref19] Li Z. Li X. Zeng Q. Chen M. Liu D. Wang J.. (2018). Genome sequence of *Pseudomonas chlororaphis* Lzh-T5, a plant growth-promoting rhizobacterium with antimicrobial activity. Genome Announc. 6:e00328-18. doi: 10.1128/genomeA.00328-18, PMID: 29724833 PMC5940960

[ref20] Li S. Yue S. J. Huang P. Feng T. T. Zhang H. Y. Yao R. L. H.. (2022). Comparative metabolomics and transcriptomics analyses provide insights into the high-yield mechanism of phenazines biosynthesis in *Pseudomonas chlororaphis* GP72. J. Appl. Microbiol. 133, 2790–2801. doi: 10.1111/jam.15727, PMID: 35870153

[ref21] Li X. J. Zhang W. Zhao C. N. Wu Q. L. Li J. K. Xu Z. H. (2021). Synthesis and fungicidal activity of phenazine-1-carboxylic triazole derivatives. J. Asian Nat. Prod. Res. 23, 452–465. doi: 10.1080/10286020.2020.1754400, PMID: 32378430

[ref22] Liu K. Hu H. Wang W. Zhang X. (2016). Genetic engineering of *Pseudomonas chlororaphis* GP72 for the enhanced production of 2-hydroxyphenazine. Microb. Cell Fact. 15:131. doi: 10.1186/s12934-016-0529-0, PMID: 27470070 PMC4965901

[ref23] Liu K. Li L. Yao W. Wang W. Huang Y. Wang R.. (2021). Genetic engineering of *Pseudomonas chlororaphis* Lzh-T5 to enhance production of trans-2,3-dihydro-3-hydroxyanthranilic acid. Sci. Rep. 11:16451. doi: 10.1038/s41598-021-94674-8, PMID: 34385485 PMC8361184

[ref24] Livak K. J. Schmittgen T. D. (2001). Analysis of relative gene expression data using real-time quantitative PCR and the 2(-Delta Delta C(T)) method. Methods 25, 402–408. doi: 10.1006/meth.2001.1262 11846609

[ref25] Lugtenberg B. Kamilova F. (2009). Plant-growth-promoting rhizobacteria. Annu. Rev. Microbiol. 63, 541–556. doi: 10.1146/annurev.micro.62.081307.162918 19575558

[ref26] Mavrodi D. V. Blankenfeldt W. Thomashow L. S. (2006). Phenazine compounds in *Fluorescent pseudomonas* spp. biosynthesis and regulation. Annu. Rev. Phytopathol. 44, 417–445. doi: 10.1146/annurev.phyto.44.013106.145710 16719720

[ref27] Mavrodi D. V. Bonsall R. F. Delaney S. M. Soule M. J. Phillips G. Thomashow L. S. (2001). Functional analysis of genes for biosynthesis of pyocyanin and phenazine-1-carboxamide from *Pseudomonas aeruginosa* PAO1. J. Bacteriol. 183, 6454–6465. doi: 10.1128/JB.183.21.6454-6465.2001, PMID: 11591691 PMC100142

[ref28] Medina M. B. Resnik S. L. Munitz M. S. (2021). Optimization of a rice cooking method using response surface methodology with desirability function approach to minimize pesticide concentration. Food Chem. 352:129364. doi: 10.1016/j.foodchem.2021.129364, PMID: 33657482

[ref29] Meng Z. Chen X. Guan L. Xu Z. Zhang Q. Song Y.. (2018). Dissipation kinetics and risk assessments of tricyclazole during *Oryza sativa* L. growing, processing and storage. Environ. Sci. Pollut. Res. Int. 25, 35249–35256. doi: 10.1007/s11356-018-3445-5, PMID: 30341752

[ref30] Molina J. Sikora M. Garud N. Flowers J. M. Rubinstein S. Reynolds A.. (2011). Molecular evidence for a single evolutionary origin of domesticated rice. Proc. Natl. Acad. Sci. U. S. A. 108, 8351–8356. doi: 10.1073/pnas.1104686108, PMID: 21536870 PMC3101000

[ref31] Olyaei A. Sadeghpour M. (2022). A review on lawsone-based benzo[a]phenazin-5-ol: synthetic approaches and reactions. RSC Adv. 12, 13837–13895. doi: 10.1039/D2RA02139K, PMID: 35541431 PMC9082651

[ref32] Padovani L. Capri E. Padovani C. Puglisi E. Trevisan M. (2006). Monitoring tricyclazole residues in rice paddy watersheds. Chemosphere 62, 303–314. doi: 10.1016/j.chemosphere.2005.05.025, PMID: 15996714

[ref33] Parsons J. F. Greenhagen B. T. Shi K. Calabrese K. Robinson H. Ladner J. E. (2007). Structural and functional analysis of the pyocyanin biosynthetic protein PhzM from *Pseudomonas aeruginosa*. Biochemistry 46, 1821–1828. doi: 10.1021/bi6024403, PMID: 17253782 PMC2572083

[ref34] Peng H. Zhang P. Bilal M. Wang W. Hu H. Zhang X. (2018). Enhanced biosynthesis of phenazine-1-carboxamide by engineered *Pseudomonas chlororaphis* HT66. Microb. Cell Fact. 17:117. doi: 10.1186/s12934-018-0962-3, PMID: 30045743 PMC6060551

[ref35] Petitjean M. Juarez P. Meunier A. Daguindau E. Puja H. Bertrand X.. (2021). The rise and the fall of a *Pseudomonas aeruginosa* endemic lineage in a hospital. Microb. Genom. 7:000629. doi: 10.1099/mgen.0.000629 34473016 PMC8715434

[ref36] Rodriguez A. Martinez J. A. Flores N. Escalante A. Gosset G. Bolivar F. (2014). Engineering *Escherichia coli* to overproduce aromatic amino acids and derived compounds. Microb. Cell Fact. 13:126. doi: 10.1186/s12934-014-0126-z, PMID: 25200799 PMC4174253

[ref37] Sefiloglu F. O. Tezel U. Balcioglu I. A. (2021). Validation of an analytical workflow for the analysis of pesticide and emerging organic contaminant residues in paddy soil and rice. J. Agric. Food Chem. 69, 3298–3306. doi: 10.1021/acs.jafc.0c06111, PMID: 33427464

[ref38] Sharma A. Shukla A. Attri K. Kumar M. Kumar P. Suttee A.. (2020). Global trends in pesticides: a looming threat and viable alternatives. Ecotoxicol. Environ. Saf. 201:110812. doi: 10.1016/j.ecoenv.2020.110812, PMID: 32512419

[ref39] Sun X. Xu Y. Chen L. Jin X. Ni H. (2021). The salt-tolerant phenazine-1-carboxamide-producing bacterium *Pseudomonas aeruginosa* NF011 isolated from wheat rhizosphere soil in dry farmland with antagonism against *Fusarium graminearum*. Microbiol. Res. 245:126673. doi: 10.1016/j.micres.2020.126673, PMID: 33429287

[ref40] Takeuchi K. Tsuchiya W. Noda N. Suzuki R. Yamazaki T. Haas D. (2014). Lon protease negatively affects GacA protein stability and expression of the Gac/Rsm signal transduction pathway in *Pseudomonas protegens*. Environ. Microbiol. 16, 2538–2549. doi: 10.1111/1462-2920.12394, PMID: 24428244

[ref41] Talapatra S. Talapatra B. (2005). Shikimic acid pathway. Berlin, Germany: Springer.

[ref42] Walker J. Moore G. (2015). *Pseudomonas aeruginosa* in hospital water systems: biofilms, guidelines, and practicalities. J. Hosp. Infect. 89, 324–327. doi: 10.1016/j.jhin.2014.11.019, PMID: 25623205

[ref43] Wang D. Lee S. H. Seeve C. Yu J. M. Pierson L. S. 3rd Pierson E. A. (2013). Roles of the Gac-Rsm pathway in the regulation of phenazine biosynthesis in *Pseudomonas chlororaphis* 30-84. Microbiology 2, 505–524. doi: 10.1002/mbo3.90, PMID: 23606419 PMC3684763

[ref44] Whistler C. A. Stockwell V. O. Loper J. E. (2000). Lon protease influences antibiotic production and UV tolerance of *Pseudomonas fluorescens* Pf-5. Appl. Environ. Microbiol. 66, 2718–2725. doi: 10.1128/AEM.66.7.2718-2725.2000, PMID: 10877760 PMC92065

[ref45] Yan B. Ye F. Gao D. (2015). Residues of the fungicide epoxiconazole in rice and paddy in the Chinese field ecosystem. Pest Manag. Sci. 71, 65–71. doi: 10.1002/ps.3763, PMID: 24550150

[ref46] Yue S. Bilal M. Guo S. Hu H. Wang W. Zhang X. (2018). Enhanced trans-2,3-dihydro-3-hydroxyanthranilic acid production by pH control and glycerol feeding strategies in engineered *Pseudomonas chlororaphis* GP72. J. Chem. Technol. Biotechnol. 93, 1618–1626. doi: 10.1002/jctb.5531

[ref47] Zhao W. Chou J. Li J. Xu Y. Li Y. Hao Y. (2022). Impacts of extreme climate events on future rice yields in global major rice-producing regions. Int. J. Environ. Res. Public Health 19:4437. doi: 10.3390/ijerph19084437 35457305 PMC9031651

[ref48] Zhou L. Jiang H. X. Sun S. Yang D. D. Jin K. M. Zhang W.. (2016). Biotechnological potential of a rhizosphere *Pseudomonas aeruginosa* strain producing phenazine-1-carboxylic acid and phenazine-1-carboxamide. World J. Microbiol. Biotechnol. 32:50. doi: 10.1007/s11274-015-1987-y, PMID: 26873561

