# Supplementary material for: Biosynthesis and genetic engineering of phenazine-1-carboxylic acid in Pseudomonas chlororaphis Lzh-T5
Source: Front Microbiol. 2023 Apr 24;14:1186052. doi: 10.3389/fmicb.2023.1186052 (PMC10165110; doi:10.3389/fmicb.2023.1186052)
Supplement: Supplementary file 1 [file Table_1.docx]

**Frontiers in Microbiology**

**Biosynthesis and Genetic Engineering of Phenazine-1-Carboxylic Acid in *Pseudomonas chlororaphis* Lzh-T5**

Kaiquan Liu^1#^, Zhenghua Li ^1,2#^, Xiaoli Liang^1^, Yanpeng Xu^1^, Yufei Cao^1^, Ruiming Wang^1^, Piwu Li^1^, Ling Li^1,3*^

^1^State Key Laboratory of Biobased Material and Green Papermaking (LBMP), School of Bioengineering, Qilu University of Technology (Shandong Academy of Sciences), Jinan 250353, People’s Republicof China.

^2^Shandong Provincial Key Laboratory of Biophysics, Institute of Biophysics, Dezhou University, Dezhou, 253023，People’s Republic of China.

^3^Shandong Provincial Key Laboratory of Applied Microbiology, Ecology Institute, Qilu Universityof Technology (Shandong Academy of Sciences), Jinan, 250103, People’s Republic of China.

# Co-first authors have contributed equally to this work

The corresponding author: Ling Li, E-mail: liling33802400@qlu.edu.cn

The first author: Kaiquan Liu, E-mail: liukq@qlu.edu.cn

**Table S1. Primers used in this work.**

| Primers | Sequence 5’ **→** 3’ | Application |
| --- | --- | --- |
| phzF-F1 | CCGAATTCAAAGCCGGGCCAAGGCCACTGGCC | *PhzF* gene insertion |
| phzF-R1 | CGTAGTTGTGCATGGTTATCTCCCGG |  |
| PhzF-F2 | ACCATGCACAACTACGATCGTTCTATGAACA |  |
| phzF-R2 | ATAAGCTTAGCAGCCAGGCACTTCAAAGCATGT |  |
| phzO-F1 | GTTGAATTCCTTCGACACGCATCGTGGTGATCAGTG | *phzO* gene deletion |
| phzO-R1 | GGTAGCAGCCTCAGTAATGTCTAACTTTGTTTGTGTC |  |
| phzO -F2 | ATTACTGAGGCTGCTACCACCTGATTGCCGTGTAGG |  |
| phzO-R2 | TAAGGATCCCCGAACCGCCTTTGCGCAACAT |  |
| rsmE-F1 | CAACTCTAGATGCGCGACGCGGTGCTCGAT | *rsmE* gene deletion |
| rsmE-R1 | GATCTTCTCCTTGATTGCTTTGTAGGGCACCTG |  |
| rsmE-F2 | AATCAAGGAGAAGATCGCGTCATGAGCGGCCA |  |
| rsmE-R2 | GGGGAAGCTTGCTCGAAATATTGCCGATGGTG |  |
| parS-F1 | CGGGATCCCGATGGATAACCCGGGTCTTGG | *parS* gene deletion |
| parS-R1 | CTAGAGCTCCCACGCGAATG |  |
| parS-F2 | CATTCGCGTGGGAGCTCTAGAGGTCGAGGTGACGGA |  |
| parS-R2 | CCCAAGCTTGACTTCTGACGCTATCGCGGAAC |  |
| lon-F1 | GCGTCTAGAATCCACCACCAGCCAGTCCA | *lon* gene deletion |
| lon-R1 | CTCATGGGGCACCTGCGCAATGGG |  |
| lon-F2 | AGGTGCCCCATGAGACGCAGACCTGTAG |  |
| lon-R2 | GCTAAGCTTCGCCGAGCAGGGAGAACAACA |  |
| ppsA-F | CCCCTTGGTAGAGTACGTAGTTTCCCTC | *ppsA* gene cloning |
| ppsA-R | TTAGACCGCACCCTGCCCCTC |  |
| tktA-F | AAAATGCCAAGCCGTCGTGAGCGTG | *tktA* gene cloning |
| tktA-R | TTAGTCTTCCAGCAGCTCTTCAGCCTGA |  |
| ppsA-F1 | CAGCGTCTAGAATCCACCACCAGCCAGTCCA | *ppsA gene insertion* |
| ppsA-R1 | GAGGGAAACTACGTACTCTACCAAGGGG |  |
| ppsA -F2 | GAGGGGCAGGGTGCGGTCTAA |  |
| ppsA -R2 | CAGCTAAGCTTCGCCGAGCAGGGAGAACAACA |  |
| tktA-F1 | GGCGGGATCCCGATGGATAACCCGGGTCTTGG | *ppsA gene insertion* |
| tktA-R1 | CACGCTCACGACGGCTTGGCATTTT |  |
| tktA -F2 | TCAGGCTGAAGAGCTGCTGGAAGACTAA |  |
| tktA -R2 | AACCCAAGCTTGACTTCTGACGCTATCGCGGAAC |  |
| PhzF-RTF | ACGTAACTGTGGTGATGCGC | *quantitative RT-PCR analysis of phzF* |
| PhzF-RTR | TCGGTATCGGCTGGTCCATGCT |  |
| tktA- RTF | TCACCATCGACGACCTGAAG | *quantitative RT-PCR analysis of tktA* |
| tktA- RTR | CAGGAACACGTAGGTGTGGT |  |
| ppsA- RTF | GTCTTCGCTTCCCTGTTCAA | *quantitative RT-PCR analysis of ppsA* |
| PpsA-RTR | AACGACGGTTTCACCCAGGC |  |
